# Cochlear Implantation and Facial Nerve Synkinesis in an Infant with Hypoplastic Internal Auditory Canals — A Case Report

**DOI:** 10.7759/cureus.5420

**Published:** 2019-08-18

**Authors:** Jordan S Grauer, Stephen Z Shapiro, Samuel T Ostrower

**Affiliations:** 1 Otolaryngology, Charles E. Schmidt College of Medicine, Florida Atlantic University, Boca Raton, USA; 2 Neurosurgery, Charles E. Schmidt College of Medicine, Florida Atlantic University, Boca Raton, USA; 3 Pediatric Otolaryngology, Joe Dimaggio Children’s Hospital, Hollywood, USA

**Keywords:** facial nerve palsy, internal auditory canal stenosis, cochlear implantation, synkinesis, hypoplastic internal auditory canal, salmonella enteritidis

## Abstract

Sensorineural hearing loss (SNHL) is a common finding in cases of the congenital internal acoustic canal (IAC) stenosis. Previous reports reveal a relationship between IAC stenosis and facial palsy as well as vestibular dysfunction. This case identifies a patient with bilateral profound SNHL, bilateral IAC stenosis, and temporary unilateral facial palsy who went on to receive bilateral cochlear implants (CI). The facial nerve synkinesis that was found in this patient with hypoplastic IACs occurred after a cochlear implant activation. The synkinesis was ipsilateral to prior transient facial palsy after salmonella infection. Patients with IAC stenosis and cochlear nerve hypoplasia may respond well to cochlear implantation, but caution should be used when considering CI with an emphasis on counseling for possible facial nerve complications.

## Introduction

Sensorineural hearing loss (SNHL) is a common finding in cases of the congenital internal acoustic canal (IAC) stenosis. Previous reports reveal a relationship between IAC stenosis and facial palsy as well as vestibular dysfunction [1-3]. The incidence of facial nerve activation in cochlear implantation ranges from 1% to 14.9% [4-6]. However, the vast majority of studies on synkinesis in cochlear implantation were in the adult population focusing on head trauma and otosclerosis [4-5, 7]. This case identifies a patient with bilateral profound SNHL, bilateral IAC stenosis, and temporary unilateral facial palsy who went on to receive bilateral cochlear implants (CI). Clinical, radiological and surgical findings are described in this case report.

## Case presentation

**Figure 1:**
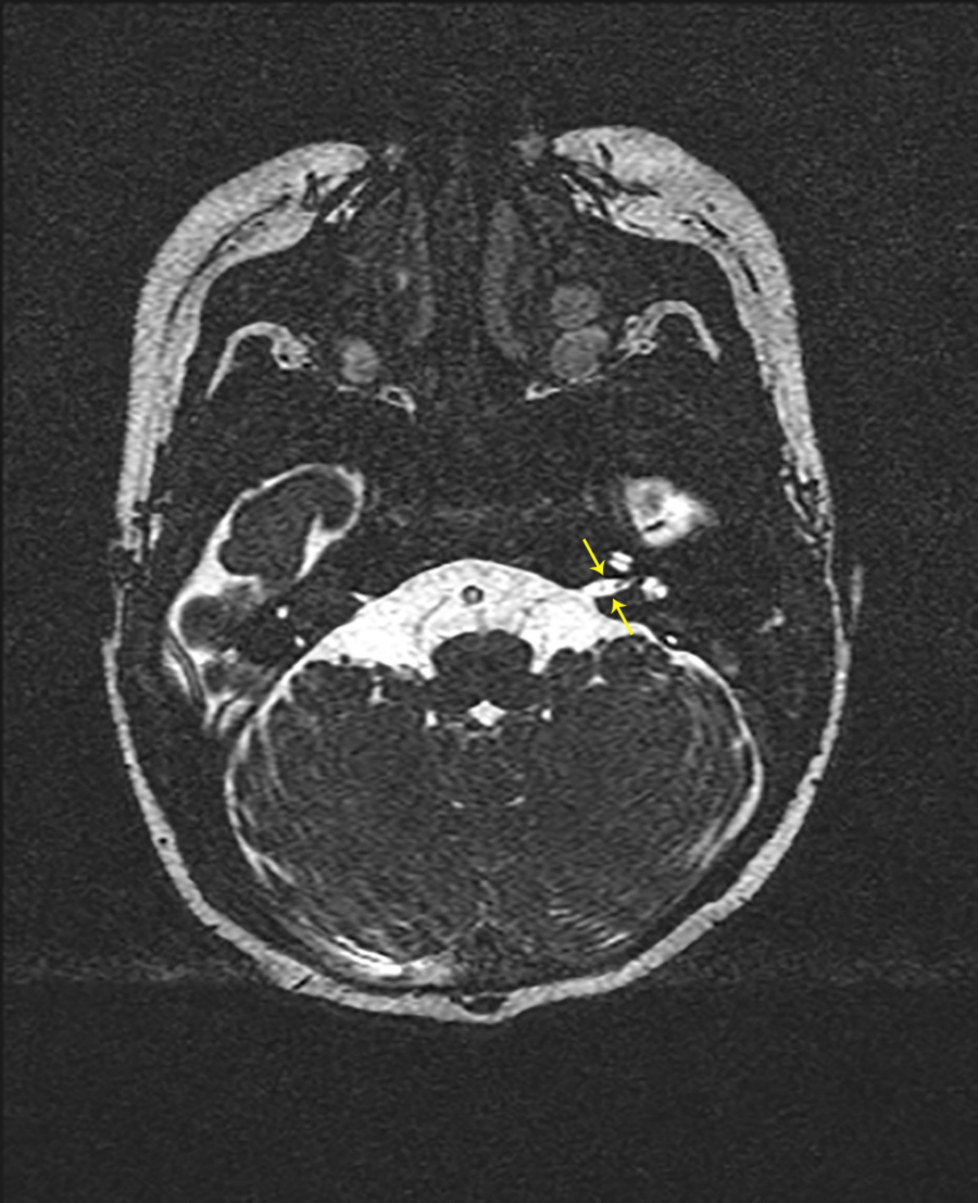
MRI of the internal auditory canals without IV contrast. CISS sequence, axial cut revealing stenotic left IAC (yellow arrows) without visible CN VIII. MRI - magnetic resonance imaging IV - intravenous CISS - constructive interference in steady state IAC - internal acoustic canal CN VIII - cranial nerve VIII (cochlear nerve)

**Figure 2:**
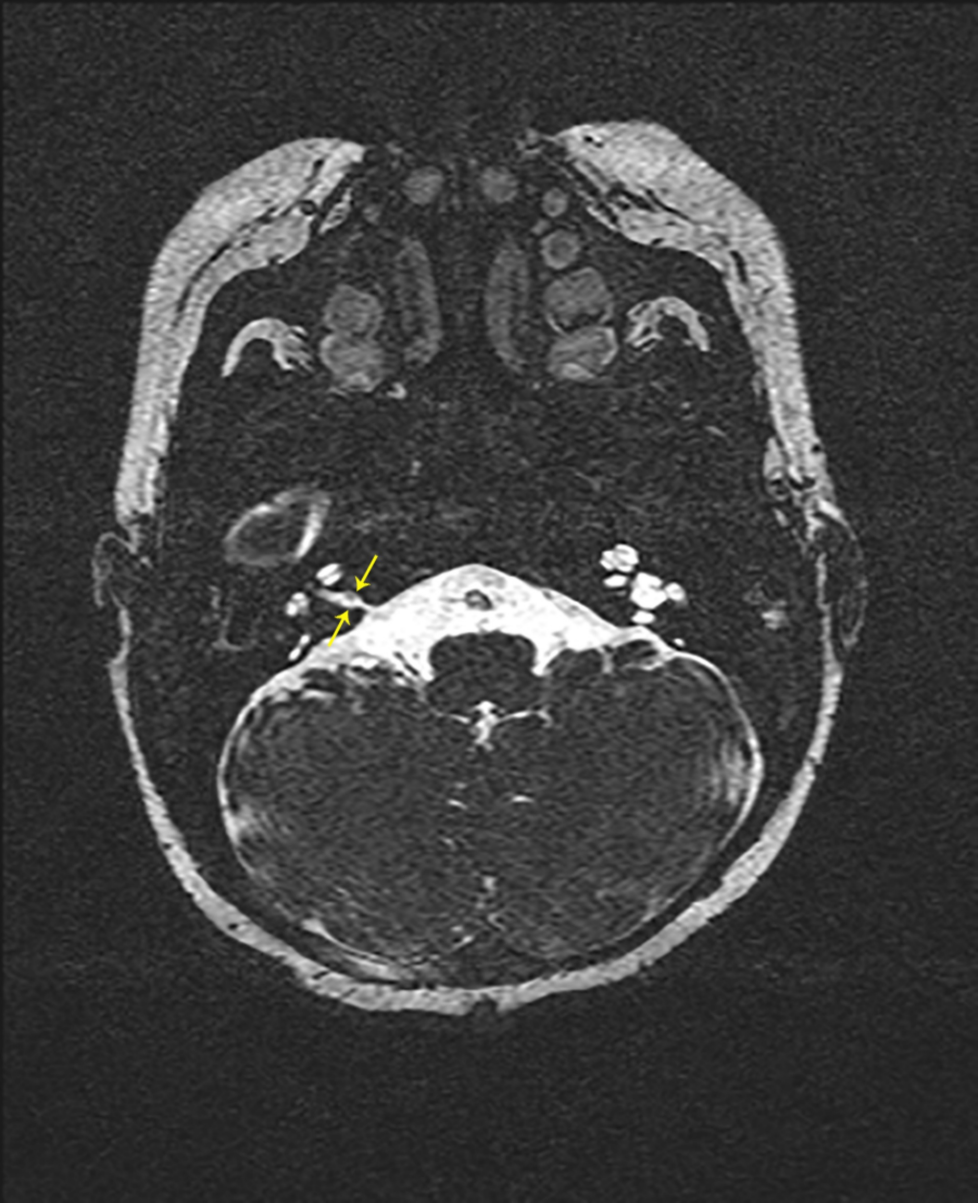
MRI of the internal auditory canals without IV contrast. CISS sequence, axial cut revealing stenotic right IAC (yellow arrows) without visible CN VIII. MRI - magnetic resonance imaging IV - intravenous CISS - constructive interference in steady state IAC - internal acoustic canal CN VIII - cranial nerve VIII (cochlear nerve)

A 14-week-old female presented to the otolaryngologist's office for evaluation of hearing loss. She was born at 36 weeks gestation following a pregnancy complicated by type II diabetes requiring insulin. She failed the newborn hearing screen (ABR) prior to discharge from the hospital and failed the two-week follow-up screen in her pediatrician’s office. A natural sleep auditory brainstem response (ABR) evaluation was performed at 10 weeks of age, which showed bilateral severe to profound sensorineural hearing loss, absent distortion product otoacoustic emissions, and no evidence of auditory neuropathy spectrum disorder (ANSD). There was no family history of early onset or congenital hearing loss and no other risk factors. Her physical examination was unremarkable. Non-contrast MRI of the brain and internal auditory canals was significant for hypoplastic bilateral internal acoustic canals (IACs), a hypoplastic right cochlear nerve, and a left cochlear nerve that could not be visualized (Figure 1, 2). A follow-up CT of the temporal bones confirmed narrow internal auditory canals bilaterally and normal course of bilateral facial nerves. She was cleared by pediatric ophthalmology, had normal EKG and renal ultrasound, and was found to have a normal microarray and GJB2 testing - a test for connexin 26 protein, a common genetic cause of nonsyndromic hereditary hearing loss - by pediatric genetics.

At four months of age, she was fit with binaural behind the ear hearing aids using average real-ear to coupler difference (RECD) values and fit to DSL targets. Post hearing aid fitting she was referred for auditory verbal therapy (AVT). Between four and six months of age, she developed intermittent right>left facial twitching independent of hearing aid use.

At seven months of age, she developed salmonella gastroenteritis, which was treated with intramuscular ceftriaxone. Her gastrointestinal illness improved, but within one week of developing fever and diarrhea, she was noted to have left facial droop and 4/6 left facial paralysis with healthy middle ears. A two-week prednisone taper was prescribed starting at 2 mg/kg, and she had near complete resolution of facial palsy within three weeks.

She had close audiologic follow-up and regular AVT. The hearing aid trial period indicated that the patient received little to no benefit from consistent use of bilateral hearing aids and that the use of traditional amplification was not providing the patient with adequate access to sounds across the frequency range. She met multidisciplinary cochlear implant team criteria for cochlear implantation (CI). A decision was made to implant the right ear first, due to the more prominent cochlear nerve on that side, and previous left facial palsy.

At 13 months of age (prior to right-sided cochlear implantation) she was found to have asymptomatic bilateral otitis media with effusion. She promptly underwent bilateral pressure equalizer (PE) tube placement with Paparella 1.02 mm tubes. Five days later, the patient underwent right-sided cochlear implantation (Cochlear Americas slim straight electrode CI522) with intraoperative facial nerve monitoring for three hours. The operation was performed uneventfully via a round window approach. Intraoperative impedances were acceptable on all 22 electrodes, and neural response telemetry (NRT) was completed on all 22 electrodes, with responses obtained on eight of 22 electrodes. The implant was activated three weeks postoperatively and CI mapping was performed over the following three months. CI mapping was completed using a combination of NRT responses as well as behavioral observation audiometry in the programming room (typically with two testers). Booth testing results were also incorporated into programming changes as the patient became more reliable and consistent. Data logging was consistent with > 6h of CI usage daily. Behavioral audiologic testing revealed that she reliably responded to speech at 25 dB and Ling 6 recorded stimuli were obtained between 20-25 dB. She continued with AVT, and demonstrated increasing accuracy of speech sound imitation.

At 18 months of age, the patient underwent left-sided CI (Cochlear Americas slim modiolar electrode CI532) with intraoperative facial nerve monitoring for three hours. The operation was performed uneventfully via a round window approach. Intraoperative impedances were acceptable, but NRI elicited facial nerve stimulation across the length of the electrode array from apical to basal. Her postoperative course was unremarkable. CI was activated three weeks later, and she was noted to have left facial stimulation at a level of 160CUs. A map was created based on NRT, and the patient was comfortable in live mode without facial stimulation. Follow-up booth testing revealed reliable responses to narrowband noise and Ling 6 sounds at 30 dB. Left-sided cochlear implant mapping challenges with this patient have revolved around providing as much access to sound as possible, while of course ensuring that facial stimulation does not occur. The most recent behavioral testing with the left CI in isolation yielded responses to Ling 6 sounds in the range of 20-30 dB.

The patient continues to tolerate bilateral CIs and demonstrates increased accuracy with identifying and imitating Ling 6 sounds and following basic one-step directions. She also has developed some simple words such as “hug” and “mama”.

## Discussion

Cochlear implants in patients with IAC and cochlear nerve hypoplasia has been well described in the literature. Papsin’s review of 298 patients with cochlear anomalies found 11 patients with narrowing of the IAC or cochlear canal [8]. Papsin found that children with IAC/cochlear canal stenosis have a poorer performance with CI and are more difficult to map. Despite these challenges, the author asserts that anomalous cochleovestibular anatomy should not be a major factor in CI candidacy.

While other authors have identified cases of congenital facial nerve palsy with IAC stenosis, acquired facial nerve palsy secondary to infectious etiology has not been presented in the literature [3, 9]. Geiseman et al. found facial nerve palsy occurrence in two of nine ears with IAC atresia and considered IAC atresia with the absence of the vestibulocochlear nerve to be a definitive contraindication to CI implantation [3]. These authors suggested that anatomic anomalies of facial nerve development led to facial nerve palsy. In our case of facial palsy and subsequent facial nerve synkinesis with a stenotic IAC, the neurologic abnormalities coincided with the patient’s salmonella infection. Cochlear implantation in this population may carry an increased risk of facial nerve complications. For further study, high fidelity MRI can be used to identify the course of the facial nerve in this patient to compare these findings to those described by Giesman et al. 

Facial nerve activation in cases of CI is a well-known complication with an incidence ranging from 1% to 14.9% [4-6]. Facial nerve synkinesis can lead to the reduced utility of CIs and effect outcomes [4]. In this patient, CI mapping was adjusted to eliminate facial nerve synkinesis during CI activation.

Patients with IAC stenosis and cochlear nerve hypoplasia may respond well to cochlear implantation, but caution should be used when considering CI with a history of ipsilateral facial palsy.

## Conclusions

In this case report, a patient with bilateral profound SNHL, bilateral IAC stenosis, and temporary unilateral facial palsy received bilateral cochlear implants, which resulted in facial nerve synkinesis after a cochlear implant activation. Patients with IAC stenosis and cochlear nerve hypoplasia may respond well to cochlear implantation, but caution should be used when considering CI with a history of ipsilateral facial palsy.
